# Effect of surgery on overall survival and cancer-specific survival in patients with primary HCC: A study based on PSM in the SEER cohort

**DOI:** 10.1097/MD.0000000000041521

**Published:** 2025-02-21

**Authors:** Lin Xia, Shuai-Xin Yu, Yu-Shuai Bai, Xiao Liang, Fu-Gui Wu, Yang Gao, Xiu-Li Chen, Zhao-Xiong Xiao, Man Li

**Affiliations:** a School of Public Health, Hebei Medical University, Shijiazhuang, Hebei, China; b Shijiazhuang Fifth Hospital, Shijiazhuang, Hebei, China; c Cangzhou Central Hospital, Cangzhou, Hebei, China.

**Keywords:** hepatocellular carcinoma, liver transplantation, propensity score matching, SEER, surgery

## Abstract

This study was designed to assess the effects of surgery method on overall survival (OS) and cancer-specific survival (CSS) in patients with hepatocellular carcinoma (HCC). This is a retrospective study. Patients diagnosed with primary HCC (N = 10,174) were identified from the Surveillance, Epidemiology, and End Results Database from 2010 to 2017 and categorized into surgical (N = 4950) and nonsurgical (N = 5224) groups. The characteristics of patients were balanced by propensity score matching. Multivariate Cox analysis was used to explore independent prognostic factors for outcomes in both groups, and the Kaplan–Meier curve showed survival rates in each group. The surgical patients were subclassified by surgical method, whether local tumor destruction, wedge or segmental resection, lobotomy resection, or liver transplantation (LT). Finally, survival rates in the 2 groups were investigated by subgroup analysis. After propensity score matching, sex, grade, tumor node metastasis III/IV, surgery, chemotherapy, alpha-fetoprotein, number of regional lymph nodes, other race, and age > 70 were independent prognostic factors in the 2 groups. The OS (HR = 0.290, *P* < .001) and CSS (HR = 0.274, *P* < .001) rates of patients were higher in the surgical group than in the nonsurgical group. There was no obvious improvement in CSS in patients who received radiotherapy combined with surgery compared with patients who only received radiotherapy (HR = 0.813, *P* = .279). LT was consistently found to be the best of the 4 surgical methods. The OS of stage II patients undergoing LT was better than that of corresponding stage III patients, and lobectomy resection was the best choice for stage IV patients (HR = 0. 417, *P* = .023). In grade III patients, the median CSS time was longer than the OS time. The survival rate of patients treated with chemotherapy combined with LT was higher than that of patients who did not receive chemotherapy and only received LT. Patients with HCC who underwent surgery had better OS and CSS. Subgroup analysis showed that LT can improve the survival rate and median survival time of patients.

## 1. Introduction

Primary liver cancer is one of the most common types of malignant tumors in the world, ranking 6th in incidence and 3rd in mortality among all cancers.^[1]^ Hepatocellular carcinoma (HCC) is the most common pathological classification of primary liver cancer, accounting for 85% to 90%,^[2]^ and it is also the focus of research in primary liver cancer.

The etiology of primary HCC is complex, with factors such as hepatitis B and hepatitis C viruses, chronic alcoholism, nonalcoholic fatty liver disease (NAFLD), and metabolic factors all potentially contributing to the development of HCC.^[3–5]^ Infections with hepatitis B and hepatitis C are major risk factors for HCC. These viruses damage hepatocytes through chronic inflammation and immune responses, promoting their proliferation, and transformation into cancer. These viral infections can lead to chronic hepatitis, which may progress to cirrhosis and ultimately develop into HCC.^[6–8]^ The metabolite of alcohol, acetaldehyde, has a direct toxic effect on hepatocytes. Chronic alcoholism leads to hepatocyte damage, inflammation, and fibrosis, increasing the risk of HCC.^[9]^ With the rise in obesity and metabolic syndrome, NAFLD has become a significant risk factor for HCC.^[10]^ NAFLD causes hepatocyte damage and canceration through oxidative stress, endoplasmic reticulum stress, and metabolic disorders.^[11]^ Metabolic diseases such as type 2 diabetes promote hepatocyte canceration through insulin resistance, inflammation, and metabolic disorders.^[12]^ These factors interact through various mechanisms, collectively promoting the occurrence and progression of primary HCC.

At present, the main treatment methods for HCC are surgery, interventional therapy, radiotherapy, transarterial chemoembolization (TACE), and antitumor drug therapy.^[13,14]^ The prognosis is extremely poor due to the patient’s strong resistance to chemotherapy. TACE is mainly used in unresectable HCC patients.^[14]^ Surgical treatment is an ideal method to eradicate HCC, and patients can have a better survival rate. Surgical treatments include wedge or segmental resection (WSR), lobotomy resection (LR), liver transplantation (LT), and local tumor destruction (LTD: these include heat-radio-frequency ablation, photodynamic therapy, laser, percutaneous ethanol injection, microwave ablation, cryoablation, and so on, all of which were defined as LTD in this study). Studies have shown that surgery is a better way to treat HCC^[15–18]^ and can improve the prognosis of patients with HCC.^[19–23]^ However, for patients with different stages, the preferred surgery methods are not the same.

The Surveillance, Epidemiology, and End Results Database (SEER) is the National Cancer Institute’s authoritative cancer statistics database, which includes medical information on nearly one-third of the U.S. population and is an important platform for cancer research.^[24]^ The propensity score can balance the baseline characteristics of the 2 groups and exclude the influence of confounding variables to make the results more accurate. In this study, the propensity score matching (PSM) method was used to explore the overall survival (OS) rate and cancer-specific survival (CSS) rate of patients with primary HCC after different surgical methods based on the SEER database to provide evidence for clinical surgical treatment. No such studies have been conducted before.

## 2. Methods

### 2.1. Patients and data collection

The study enrolled patients from the American Institute for Cancer Research SEER database. The SEER database is a public database commonly used in clinical practice that contains a large amount of clinical tumor retrospective data, including patient demographics, primary tumor site, tumor morphology, tumor stage, treatment, and survival status. Data from the SEER database are publicly available and comply with the Declaration of Helsinki. Therefore, it can be applied directly without the need for ethical review. The patient data were obtained by downloading the data to SEER-Stat (version 8.3.5) software.

**Figure 1.**
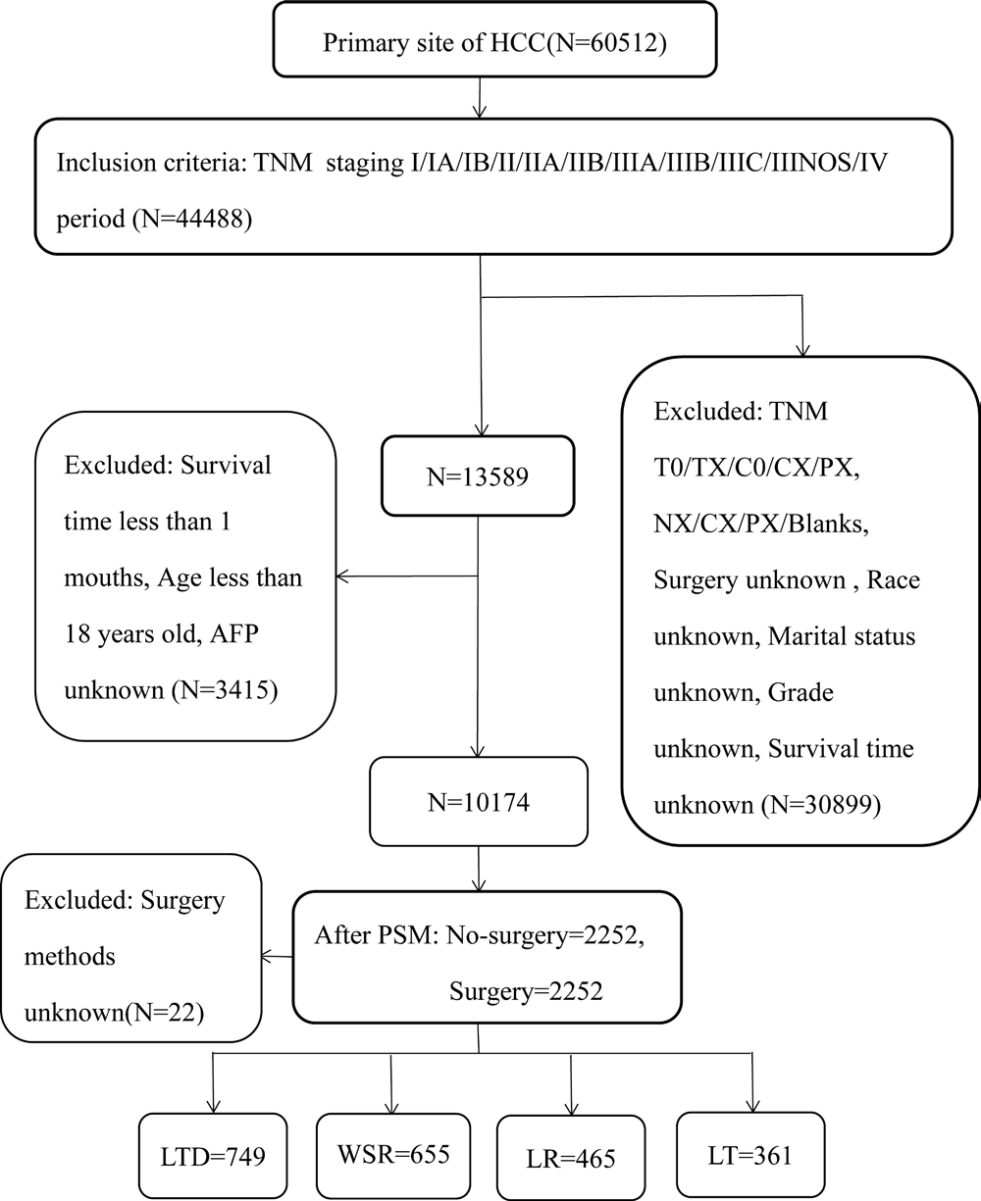
Flow diagram of HCC patient selection from the SEER database. HCC = hepatocellular carcinoma, SEER = Surveillance, Epidemiology, and End Results Database.

As shown in Figure 1, we selected 60,512 patients diagnosed with primary liver cancer from 2010 to 2017 from the SEER database based on the International Classification of Tumor Diseases code. Next, we included tumor node metastasis (TNM) stage I/IA/IB/II/IIA/IIB/IIIA/IIIB/IIIC/IIINOS/IV patients, excluding T0/TX/C0/CX/PX, NX/CX/PX/Blanks, and not appropriate patients. In addition, patients with unknown surgery, unknown marital status, unknown race, unknown grade, unknown survival time, and unknown alpha-fetoprotein (AFP) levels before treatment were excluded from the target population. We also excluded patients younger than 18 years of age and those with survival times of <1 month.

### 2.2. Variables and classification

The variables included in this study included age, sex, histotype, AFP level before treatment, race, marital status, TNM total stage, T stage, N stage, surgery, radiotherapy, chemotherapy, number of regional lymph nodes, pathological grade, first malignant primary indicator (primary), survival time, and survival outcome. The detailed classification of the research variables is shown in Table S1, Supplemental Digital Content, http://links.lww.com/MD/O428.

### 2.3. Definition of outcome indicators

The results mainly consist of 2 parts: the OS outcome of HCC and the CSS outcome of HCC. The OS of HCC refers to the time from diagnosis of HCC to death from any cause (the last follow-up time was recorded for the patients who were lost to follow-up, and the patients who were still alive at the end of the study were the end date of follow-up). The CSS refers to the time of death caused by HCC.

### 2.4. Propensity score matching

In addition, we used propensity scores to match baseline characteristics between the surgery and no-surgery groups, excluding confounding factors. The outcome indicators of the 2 groups were matched 1:1 to balance the study variables, and the caliper value was 0.03. Variables included in PSM include age, sex, histotype, AFP, race, marital status, TNM total stage, T stage, N stage, radiotherapy, chemotherapy, number of regional lymph nodes, grade, and primary. In this study, *P*<.05 (bilateral test) was considered significant.

### 2.5. Statistical analysis

In this study, we mainly used R (version 4.2.1) software for statistical analysis. We converted continuous variables such as age and number of regional lymph nodes into categorical variables. The Chi-square test was used for categorical variables, and the *T* test was used for continuous variables to compare the basic pathological characteristics of the surgical and nonsurgical groups. Univariate and multivariate Cox regression analyses were performed for both outcomes to assess HR values and determine 95% confidence intervals. Finally, Kaplan–Meier curves were drawn for each group, and differences in survival time were compared by the log-rank test.

## 3. Results

### 3.1. Clinical pathological features

A total of 10,174 patients diagnosed with primary HCC were included in the study, including 4950 patients in the surgical group and 5224 patients without surgery. The 2 groups had significant differences in sex, grade, TNM stage, radiotherapy, chemotherapy, AFP, primary, race, age, marriage, and regional lymph node number before PSM. Nearly half (48.4%) of the patients enrolled from 2010 to 2017 in the SEER database were grade II. The proportions of patients receiving chemotherapy (54.7% vs 23.9%), radiotherapy (16.7% vs 3.5%), and exhibiting AFP positivity were higher in the nonsurgery group than in the surgery group. The proportion of patients receiving chemotherapy was higher than that receiving radiotherapy in both the surgery group (23.9% vs 3.5%) and the nonsurgery group (54.7% vs 16.7%). More detailed results are shown in Table 1.

**Table 1:** Clinical characteristics of the study population.

Variables (%)	Before PSM				After PSM			
	Total (10,174)	No-surgery (5224)	Surgery (4950)	*P*	Total (4504)	No-surgery (2252)	Surgery (2252)	*P*
Sex				<.001				.170
** **Female	2445 (24.0)	1125 (21.5)	1320 (26.7)		1136 (25.2)	548 (24.3)	588 (26.1)	
** **Male	7729 (76.0)	4099 (78.5)	3630 (73.3)		3368 (74.8)	1704 (75.7)	1664 (73.9)	
Grade				<.001				.925
** **Ⅰ	3030 (29.8)	1740 (33.3)	1290 (26.1)		1373 (30.5)	685 (30.4)	688 (30.6)	
** **Ⅱ	4920 (48.4)	2247 (43.0)	2673 (54.0)		2158 (47.9)	1078 (47.9)	1080 (48.0)	
** **Ⅲ	2066 (20.3)	1152 (22.0)	914 (18.5)		902 (20.0)	456 (20.2)	446 (19.8)	
** **Ⅳ	158 (1.6)	85 (1.6)	73 (1.5)		71 (1.6)	33 (1.5)	38 (1.7)	
Histotype				<.001				1.000
** **HCC others	189 (1.9)	74 (1.4)	115 (2.3)		79 (1.8)	37 (1.6)	42 (1.9)	
** **HCC NOS	9985 (98.1)	5150 (98.6)	4835 (97.7)		4425 (98.2)	2215 (98.4)	2210 (98.1)	
TNM				<.001				.283
** **Ⅰ	4462 (43.9)	1685 (32.3)	2777 (56.1)		2234 (49.6)	1132 (50.3)	1102 (49.0)	
** **Ⅱ	2285 (22.5)	833 (15.9)	1452 (29.3)		1150 (25.5)	564 (25.0)	586 (26.0)	
** **Ⅲ	2111 (20.7)	1530 (29.3)	581 (11.7)		872 (19.4)	421 (18.7)	451 (20.0)	
** **Ⅳ	1316 (12.9)	1176 (22.5)	140 (28.3)		248 (5.5)	135 (6.0)	113 (5.0)	
T stage				<.001				.561
** **T1	4784 (47.0)	1967 (37.7)	2817 (56.9)		2306 (51.2)	1169 (51.9)	1137 (50.5)	
** **T2	2484 (24.4)	998 (19.1)	1486 (30.0)		1201 (26.7)	589 (26.1)	612 (27.2)	
** **T3	2546 (25.0)	2020 (38.7)	526 (10.6)		850 (18.9)	427 (19.0)	423 (18.8)	
** **T4	360 (3.5)	239 (4.6)	121 (2.4)		147 (3.3)	67 (3.0)	80 (3.6)	
N stage				<.001				.355
** **N0	9479 (93.2)	4600 (88.1)	4879 (98.6)		4384 (97.3)	2187 (97.1)	2197 (97.6)	
** **N1	695 (6.8)	624 (11.9)	71 (1.4)		120 (2.7)	65 (2.9)	55 (2.4)	
Radiotherapy				<.001				.642
** **No/unknown	9131 (89.7)	4353 (83.3)	4778 (96.5)		4186 (92.9)	2089 (92.8)	2097 (93.1)	
** **Yes	1043 (10.3)	871 (16.7)	172 (3.5)		318 (7.1)	163 (7.2)	155 (6.9)	
Chemotherapy				<.001				.649
** **No/unknown	6130 (60.3)	2365 (45.3)	3765 (76.1)		2661 (59.1)	1323 (58.7)	1338 (59.4)	
** **Yes	4044 (39.7)	2859 (54.7)	1185 (23.9)		1843 (40.9)	929 (41.3)	914 (40.6)	
AFP				<.001				.575
** **Negative	3571 (35.1)	1528 (29.2)	2043 (41.3)		1592 (35.3)	787 (34.9)	805 (35.7)	
** **Positive	6603 (64.9)	3696 (70.8)	2907 (58.7)		2912 (64.7)	1465 (65.1)	1447 (64.3)	
Primary				<.001				.381
** **No	1656 (16.3)	927 (17.7)	729 (14.7)		760 (16.9)	369 (16.4)	391 (17.4)	
** **Yes	8518 (83.7)	4297 (82.3)	4221 (85.3)		3744 (83.1)	1883 (83.6)	1861 (82.6)	
Race				<.001				.626
** **White	6862 (67.4)	3690 (70.6)	3172 (64.1)		3103 (68.9)	1566 (69.5)	1537 (68.3)	
** **Black	1251 (12.3)	699 (13.4)	552 (11.2)		546 (12.1)	265 (11.8)	281 (12.5)	
** **Other	2061 (20.3)	835 (16.0)	1226 (24.8)		855 (19.0)	421 (18.7)	434 (19.3)	
Age				<.001				.219
** **≤60	3527 (34.7)	1604 (30.7)	1923 (38.8)		1538 (34.1)	796 (35.3)	742 (32.9)	
** **60–70	3660 (36.0)	1774 (34.0)	1886 (38.1)		1580 (35.1)	781 (34.7)	799 (35.5)	
** >**70	2897 (28.5)	1846 (35.3)	1141 (23.1)		1386 (30.8)	675 (30.0)	711 (31.6)	
Marital				<.001				.175
** **Divorced/separated	1325 (13.0)	721 (13.8)	604 (12.2)		637 (14.1)	297 (13.2)	340 (15.1)	
** **Married	5900 (58.0)	2819 (54.0)	3081 (62.2)		2529 (56.2)	1297 (57.6)	1232 (54.7)	
** **Single/unmarried	2011 (19.8)	1118 (21.4)	893 (18.0)		935 (20.8)	461 (20.5)	474 (21.0)	
** **Windowed	938 (9.2)	566 (10.8)	372 (7.5)		403 (8.9)	197 (8.7)	206 (9.1)	
Regional nodes				<.001				.444
** **0	9365 (92.0)	5157 (98.7)	4208 (85.0)		4392 (97.5)	2200 (97.7)	2192 (97.3)	
** **More than 0	809 (8.0)	67 (1.3)	742 (15.0)		112 (2.5)	52 (2.3)	60 (2.7)	

AFP = alpha-fetoprotein, HCC = hepatocellular carcinoma, primary = first malignant primary indicator, PSM = propensity score matching, TNM = tumor node metastasis.

### 3.2. Univariate and multivariate Cox analysis of HCC patients with OS and CSS before PSM

Univariate Cox analysis of OS showed that OS outcome was significantly correlated with sex, grades III and IV, TNM total stage, T stage, N stage, surgery, radiotherapy, chemotherapy, AFP, primary, race, age, marriage, and regional lymph nodes. By incorporating meaningful variables from univariate analysis into the multivariate Cox model, we found that the survival rate of patients with HCC in the surgery group was higher (HR = 0.268, *P* < .001) as shown in Table S2, Supplemental Digital Content, http://links.lww.com/MD/O428.

Univariate Cox regression of CSS showed that CSS outcome was significantly correlated with sex, grade, TNM stage, surgery, radiotherapy, chemotherapy, AFP, race, age > 70, marriage, regional lymph node number. Multivariate Cox regression also showed that the survival rate of patients in the surgical group was higher than that in the nonsurgical group (HR = 0.247, *P* < .001) as shown in Table S2, Supplemental Digital Content, http://links.lww.com/MD/O428.

**Figure 2.**
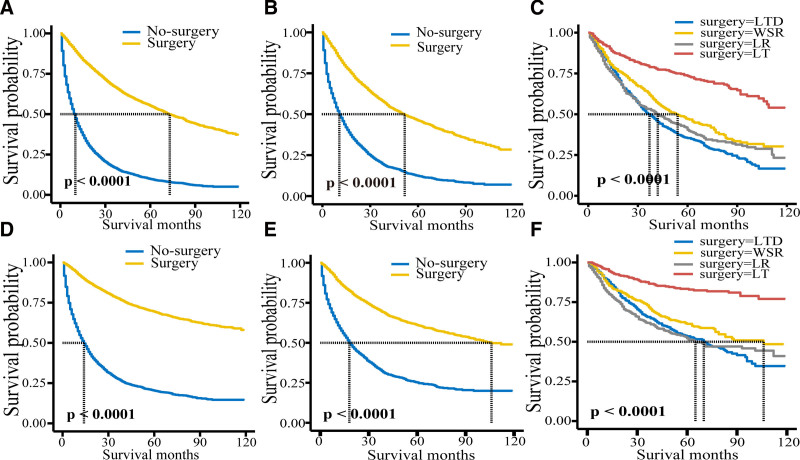
Survival analysis of patients with overall survival ((A) unmatched; (B) matched; (C) surgery methods) and specific survival ((D) unmatched; (E) matched; (F) surgery methods).

Patients who underwent surgery had better outcomes in both OS and CSS than those who did not (*P* < .001) as shown in Figure 2A and D.

### 3.3. Propensity score matching

To balance confounding factors, we performed a 1:1 PSM analysis for *P* values of significance in the included variables, and the caliper value was set to .03. The final number of patients was 4504, including 2252 patients in the surgical and nonsurgical groups. After PSM analysis, the *P* value of each covariate was more than .05 (Table 1). The LOVE graph of each categorical variable before and after matching is shown in Figure S1, Supplemental Digital Content, http://links.lww.com/MD/O428.

### 3.4. Multivariate Cox analysis of OS and CSS of patients with HCC after PSM

Multivariate analysis showed that gender, grade, TNM Ⅲ/Ⅳ, T4, surgery, chemotherapy, AFP, number of regional lymph nodes, race, age > 70, and marriage were independent prognostic factors for OS outcomes. Independent prognostic factors of CSS outcome were sex, grade, TNM Ⅲ/Ⅳ, surgery, chemotherapy, AFP, number of regional lymph nodes, race, and age > 70. Matched analysis showed that the OS (HR = 0.290, *P* < .001) and CSS (HR = 0.274, *P* < .001) rates in the surgical group were higher than those in the nonsurgical group, and patients could benefit from surgery (Table 2). Patients who received surgery had better outcomes than those who did not (*P* < .001) (Fig. 2B and E).

**Table 2:** Multivariate Cox regression analysis of overall survival and specific survival of HCC after PSM.

Variables	Overall survival of cancer		Cancer-specific survival	
	HR (95% CI)	*P* value	HR (95% CI)	*P* value
Sex				
** **Female	Reference		Reference	
** **Male	1.112 (1.021–1.212)	.015	1.040 (1.126–1.266)	.015
Grade				
** **I	Reference		Reference	
** **II	1.167 (1.072–1.270)	<.001	1.210 (1.089–1.345)	<.001
** **III	1.589 (1.432–1.763)	<.001	1.773 (1.563–2.011)	<.001
** **Ⅳ	1.822 (1.388–2.392)	<.001	1.851 (1.331–2.573)	<.001
Histotype				
** **HCC others	Reference		Reference	
** **HCC NOS	0.995 (0.763–1.299)	.973	0.938 (0.685–1.284)	.688
TNM				
** **I	Reference		Reference	
** **II	1.477 (0.997–2.189)	.052	1.414 (0.898–2.226)	.135
** **III	1.742 (1.265–2.399)	<.001	2.009 (1.384–2.916)	<.001
** **IV	2.881 (2.178–3.811)	<.001	3.303 (2.384–4.575)	<.001
T stage				
** **T1	Reference		Reference	
** **T2	0.724 (0.494–1.063)	.099	0.790 (0.508–1.227)	.294
** **T3	1.251 (0.918–1.704)	.156	1.238 (0.864–1.773)	.244
** **T4	1.431 (1.018–2.012)	.039	1.441 (0.973–2.134)	.068
N stage				
** **N0	Reference		Reference	
** **N1	1.064 (0.801–1.414)	.667	0.997 (0.718–1.385)	.987
Surgery method				
** **No-surgery	Reference		Reference	
** **Surgery	0.290 (0.269–0.312)	<.001	0.274 (0.250–0.301)	<.001
Radiotherapy				
** **No/unknown/refused	Reference		Reference	
** **Yes	0.881 (0.766–1.012)	.074	0.920 (0.779–1.086)	.325
Chemotherapy				
** **No/unknown	Reference		Reference	
** **Yes	0.735 (0.682–0.792)	<.001	0.798 (0.729–0.873)	<.001
AFP				
** **Negative/normal	Reference			
** **Positive/elevated	1.357 (1.255–1.468)	<.001	1.478 (1.341–1.630)	<.001
Region nodes				
** **0	Reference		Reference	
** **More.than.0	0.574 (0.442–0.746)	<.001	0.613 (0.453–0.829)	.001
Primary				
** **No	Reference		Reference	
** **Yes	0.923 (0.839–1.016)	1.000	1.024 (0.908–1.154)	.702
Race				
** **White	Reference		Reference	
** **Black	1.085 (0.972–1.210)	.148	1.098 (0.960–1.255)	.173
** **Others	0.834 (0.759–0.917)	<.001	0.852 (0.761–0.955)	.006
Age				
** **≤60	Reference		Reference	
** **60–70	1.076 (0.986–1.174)	.101	1.118 (1.005–1.243)	.040
** **>70	1.458 (1.325–1.604)	<.001	1.529 (1.360–1.718)	<.001
Marital				
** **Divorced/separated	Reference		Reference	
** **Married	0.848 (0.763–0.942)	.002	0.908 (0.797–1.035)	.148
** **Single/unmarried	1.068 (0.947–1.204)	.284	1.070 (0.920–1.243)	.380
** **Widowed	1.086 (0.933–1.264)	.285	1.107 (0.917–1.337)	.289

AFP = alpha-fetoprotein, HCC = hepatocellular carcinoma, Primary = first malignant primary indicator, PSM = propensity score matching, TNM = tumor node metastasis.

### 3.5. Subgroup analysis

#### 3.5.1. Analysis of surgery and no-surgery

##### 3.5.1.1. TNM stage

In the 2 groups, the survival rate in the operation group for stages Ⅰ, Ⅱ, Ⅲ, and Ⅳ was higher than that in the nonoperation group. Surgery was an independent protective factor that could reduce the risk of death (Table S3, Supplemental Digital Content, http://links.lww.com/MD/O428 and Figure S2, Supplemental Digital Content, http://links.lww.com/MD/O428).

##### 3.5.1.2. Grade stage

Multivariate analysis of the 4 grades in both groups showed that patients could benefit from surgery. Surgery was a protective factor (Table S3, Supplemental Digital Content, http://links.lww.com/MD/O428 and Figure S3, Supplemental Digital Content, http://links.lww.com/MD/O428).

##### 3.5.1.3. Radiotherapy and chemotherapy

The OS analysis showed that patients in the surgery group had a higher survival rate than those in the nonsurgery group, regardless of whether they received chemotherapy or radiotherapy. The CSS analysis showed that there was no significant improvement in patients treated with radiotherapy and surgery, so CSS in patients who had received radiotherapy should be carefully considered before surgery (Table S3, Supplemental Digital Content, http://links.lww.com/MD/O428 and Figure S4, Supplemental Digital Content, http://links.lww.com/MD/O428).

#### 3.5.2. Subgroup analysis of 4 surgical methods

The OS outcome and CSS outcome were analyzed. LT was the most effective surgical treatment, followed by LR and WSR. The median survival time for patients in both groups receiving LT was more than 10 years (Table 3 and Figure 2C and F).

**Table 3:** Multivariate Cox regression analysis of TNM stage, grade stage, radiotherapy, and chemotherapy in 2 groups of patients with different surgical methods.

Variables		Overall survival of cancer		Cancer-specific survival	
		HR (95% CI)	*P* value	HR (95% CI)	*P* value
TNM stage					
	Ⅰ				
	WSR vs LTD	0.580 (0.467–0.722)	<.001	0.615 (0.459–0.824)	.001
	LR vs LTD	0.529 (0.398–0.704)	<.001	0.605 (0.419–0.874)	.007
	LT vs LTD	0.319 (0.224–0.454)	<.001	0.239 (0.140–0.408)	<.001
	Ⅱ				
	WSR vs LTD	0.731 (0.538–0.993)	.045	0.760 (0.519–1.112)	.158
	LR vs LTD	0.726 (0.495–1.064)	.101	0.914 (0.581–1.437)	.697
	LT vs LTD	0.221 (0.150–0.325)	<.001	0.168 (0.101–0.279)	<.001
	Ⅲ				
	WSR vs LTD	0.694 (0.483–0.998)	.049	0.889 (0.580–1.363)	.589
	LR vs LTD	0.757 (0.539–1.065)	.110	0.915 (0.611–1.369)	.664
	LT vs LTD	0.458 (0.283–0.740)	.001	0.463 (0.256–0.836)	.011
	Ⅳ				
	WSR vs LTD	0.440 (0.212–0.912)	.027	0.390 (0.171–0.887)	.025
	LR vs LTD	0.417 (0.197–0.884)	.023	0.355 (0.155–0.810)	.014
	LT vs LTD	0.474 (0.175–1.287)	.143	0.355 (0.111–1.133)	.080
Grade					
	Ⅰ				
	WSR vs LTD	0.615 (0.451–0.838)	.002	0.771 (0.517–1.148)	.201
	LR vs LTD	0.483 (0.329–0.710)	<.001	0.435 (0.255–0.743)	.002
	LT vs LTD	0.314 (0.207–0.477)	<.001	0.241 (0.134–0.436)	<.001
	Ⅱ				
	WSR vs LTD	0.589 (0.475–0.731)	<.001	0.543 (0.410–0.720)	<.001
	LR vs LTD	0.618 (0.481–0.795)	<.001	0.757 (0.558–1.026)	.073
	LT vs LTD	0.293 (0.217–0.395)	<.001	0.240 (0.160–0.360)	<.001
	Ⅲ				
	WSR vs LTD	0.760 (0.546–1.059)	.105	0.888 (0.603–1.308)	.547
	LR vs LTD	0.808 (0.575–1.134)	.218	0.845 (0.566–1.262)	.411
	LT vs LTD	0.201 (0.095–0.427)	<.001	0.294 (0.149–0.579)	<.001
Radiotherapy					
	No/unknown				
	WSR vs LTD	0.635 (0.544–0.741)	<.001	0.679 (0.558–0.826)	<.001
	LR vs LTD	0.667 (0.562–0.792)	<.001	0.733 (0.593–0.907)	.004
	LT vs LTD	0.319 (0.255–0.399)	<.001	0.254 (0.187–0.344)	<.001
	Yes				
	WSR vs LTD	0.771 (0.363–1.638)	.499	1.291 (0.513–3.254)	.587
	LR vs LTD	0.303 (0.140–0.656)	.002	0.401 (0.156–1.030)	.058
	LT vs LTD	0.212 (0.093–0.481)	<.001	0.171 (0.061–0.480)	<.001
Chemotherapy					
	No/unknown				
	WSR vs LTD	0.651 (0.537–0.788)	<.001	0.634 (0.490–0.819)	<.001
	LR vs LTD	0.661 (0.531–0.823)	<.001	0.701 (0.529–0.928)	.013
	LT vs LTD	0.448 (0.320–0.626)	<.001	0.385 (0.241–0.614)	<.001
	Yes				
	WSR vs LTD	0.677 (0.527–0.870)	.002	0.840 (0.633–1.116)	.230
	LR vs LTD	0.575 (0.437–0.757)	<.001	0.618 (0.448–0.851)	.003
	LT vs LTD	0.252 (0.190–0.336)	<.001	0.211 (0.145–0.306)	<.001
All					
	WSR vs LTD	0.638 (0.550–0.740)	<.001	0.690 (0.573–0.832)	<.001
	LR vs LTD	0.632 (0.535–0.746)	<.001	0.687 (0.561–0.842)	<.001
	LT vs LTD	0.315 (0.254–0.390)	<.001	0.258 (0.193–0.344)	<.001

LR = lobotomy resection, LT = liver transplantation, LTD = local tumor destruction, TNM = tumor node metastasis, WSR = wedge or segmental resection.

##### 3.5.2.1. TNM stage

**Figure 3.**
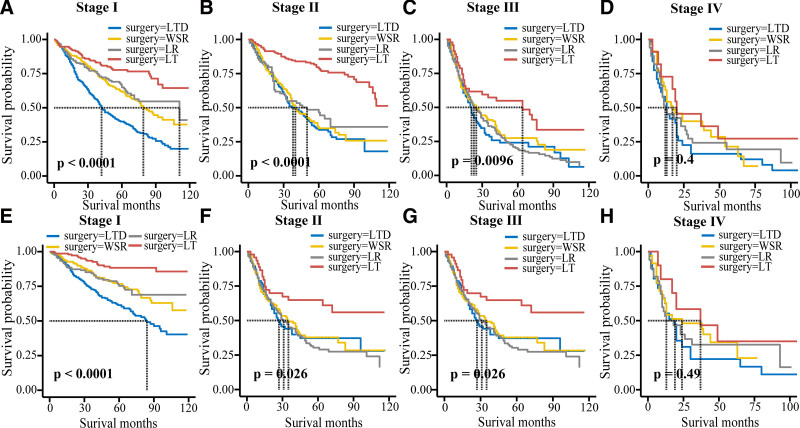
Overall survival ((A) stage Ⅰ; (B) stage Ⅱ; (C) stage Ⅲ; (D) stage Ⅳ) and specific survival ((E) stage Ⅰ; (F) stage Ⅱ; (G) stage Ⅲ; (H) stage Ⅳ) curves of patients with TNM stage of 4 surgical methods. TNM = tumor node metastasis.

The OS outcome analysis showed that the most effective surgical approach for stage I patients was LT, followed by LR and WSR. Compared with LTD, LR was not an independent prognostic factor in stage II and stage III. Patients with stage II and stage III disease had better survival after LT, but patients with stage II disease had a higher median survival time than those with stage III disease. LT was not an independent prognostic factor for stage IV patients, and LR was the best choice for stage IV patients compared to other surgical options. There was no difference in stage IV patients who underwent 4 surgical procedures by log-rank test (Table 3 and Fig. 3).

The CSS outcome analysis showed that LT was the most effective for stage I patients. WSR and LR were not independent prognostic factors in patients with stage II and stage III disease. The treatment effect of LT in patients with stage II and stage III disease is better, and the median survival time is more than 10 years. The log-rank test showed no significant difference between stage IV patients who underwent the 4 surgical methods (Table 3 and Fig. 3).

##### 3.5.2.2. Grade stage

**Figure 4.**
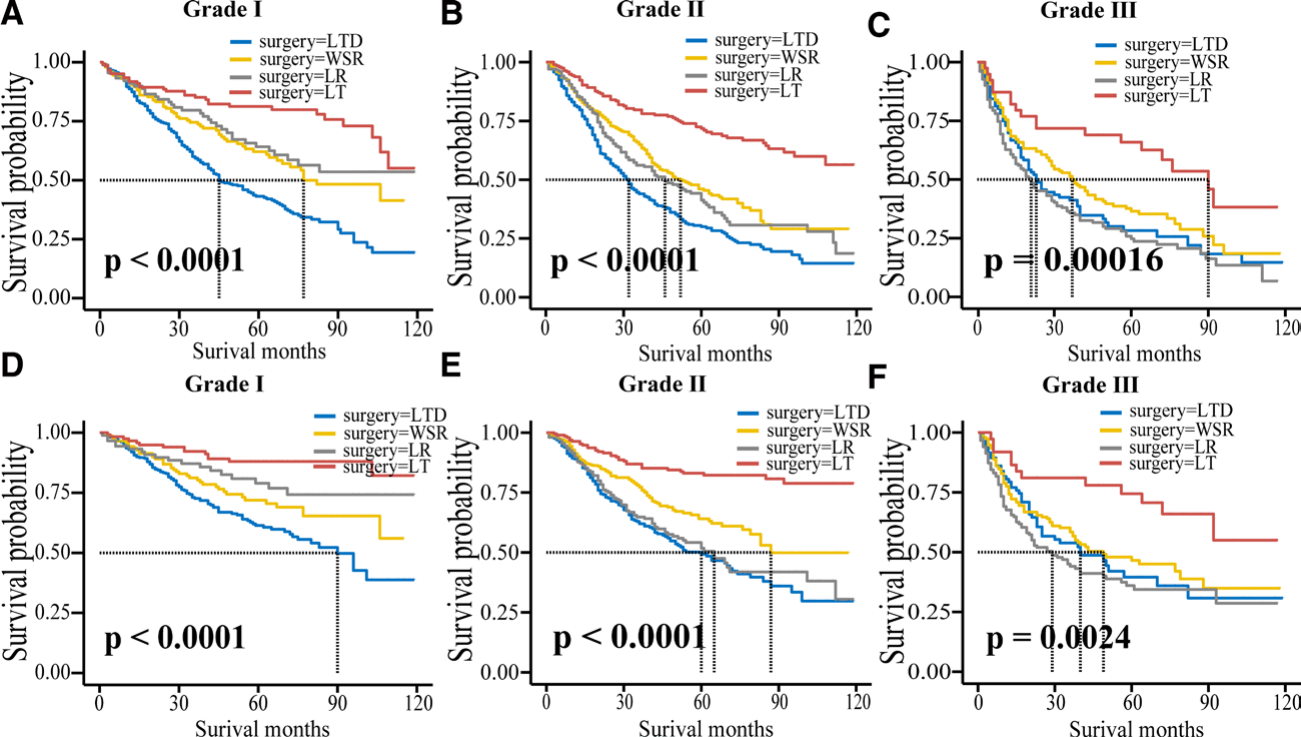
Overall survival ((A) grade Ⅰ; (B) grade Ⅱ; (C) grade Ⅲ) and specific survival ((D) grade Ⅰ; (E) grade Ⅱ; (F) grade Ⅲ) curves of patients with grade stage of 4 surgical methods.

In the analysis of OS, the most effective surgical approach for stage I patients was LT, followed by LR and WSR. The median survival time for both LR and LT is more than 10 years, so both LR and LT can be considered for stage I patients. Compared to LTD, WSR, LR, and LT were all associated with a lower risk of death in stage II patients. However, patients who received LT had longer median survival. Multivariate Cox analysis of stage III patients showed that only LT was an independent protective factor, and the median survival time was shorter for stage III patients than for patients in the first 2 stages (Table 3 and Fig. 4).

In the analysis of CSS, WSR was not an independent prognostic factor for stage I patients. Stage I patients receiving LT had the longest median survival. LR is not an independent prognostic factor in stage II patients, while WSR and LT are independent protective factors in stage II patients, but the median survival time of patients receiving WSR is shorter. Therefore, LT is the best treatment option for stage II patients. Multivariate Cox analysis of stage III patients showed that only LT was an independent protective factor. Compared with CSS of patients in the first 2 stages, the median survival time of stage III patients was shorter, but the median survival time was longer than the OS time of stage III patients. Because the sample of stage IV patients was too small to be representative, the outcome of stage IV patients in the 2 groups was not analyzed (Table 3 and Fig. 4).

##### 3.5.2.3. Radiotherapy and chemotherapy

**Figure 5.**
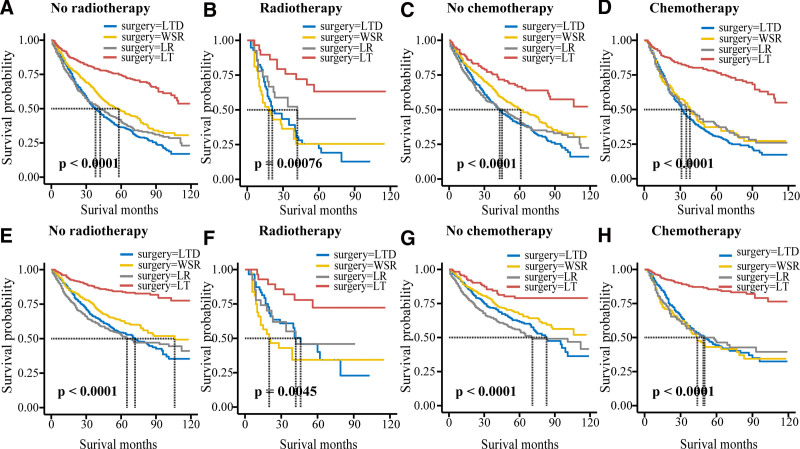
Overall survival ((A) no radiotherapy; (B) radiotherapy; (C) no chemotherapy; (D) chemotherapy) and specific survival ((E) no radiotherapy; (F) radiotherapy; (G) no chemotherapy; (H) chemotherapy) curves of patients with radiotherapy and chemotherapy of 4 surgical methods.

In the outcome of OS, we found that LT was the most effective and provided the longest median survival for patients who did not receive chemotherapy or radiotherapy. WSR is not an independent prognostic factor in patients who have received radiotherapy. LT was most effective in patients who had received chemotherapy, and the median survival time was longer than that in patients who did not receive chemotherapy. Therefore, surgery can improve the survival rate of patients who have received chemotherapy (Table 3 and Fig. 5).

For CSS outcomes, patients who did not receive chemotherapy or radiotherapy had the same outcomes as OS patients. WSR and LR are not independent prognostic factors in patients who have received radiotherapy, and only LT is effective in patients who have received radiotherapy. Patients who received chemotherapy combined with LT had a higher survival rate than those who did not receive chemotherapy and only received LT (Table 3 and Fig. 5).

## 4. Discussion

HCC is a common malignant tumor of the liver with a high malignancy degree and hidden incidence. The number of patients is increasing year by year,^[25]^ the mortality and recurrence rates are high, and the 5-year survival rate is <20%,^[26]^ which is the same as our OS outcome. Most HCC patients need to undergo surgery, which is an ideal way to eradicate HCC, and patients can have a better survival rate. Multivariate analysis showed that TNM and grade were independent prognostic factors for the outcome in the 2 groups. Chemotherapy and radiotherapy are common methods of cancer treatment. However, for HCC patients with different stages, different treatment methods have different therapeutic effects, so we performed a subgroup analysis to select appropriate surgical methods. The objective of this study was to analyze based on PSM the survival rate of HCC patients who underwent different surgical procedures at different periods.

The results showed that AFP positivity and age > 70 were independent risk factors for both OS and CSS before and after matching, which was consistent with previous studies.^[27–30]^ Preoperative AFP levels are particularly important for patients with HCC. In general, younger patients have a better ability to regenerate the liver. Senility reduces the quality of the liver, which leads to a higher risk of liver disease. Radiotherapy is not an independent prognostic factor for patients with CSS after being matched. This result indicated that radiotherapy does not affect the survival rate of patients with CSS after excluding the influence of confounding factors. Radiotherapy did not extend the survival of patients compared with no radiotherapy, which indicated that patients’ liver function might not affect the survival of patients in the current study. This is also confirmed by Huang.^[31]^ Chemotherapy and surgery were independent protective factors for outcomes in both groups. Huang study did not show that chemotherapy was beneficial for patients, possibly because Huang mainly studied independent influencing factors for large HCC, and chemotherapy had no effect on patients with tumors larger than 10 cm in size.

PSM and multivariate analysis showed that surgery could improve the survival rate of HCC patients in both groups, and the median survival time of patients with CSS was longer than that of patients with OS. Then, subgroup analysis of patients with OS and CSS showed that surgery combined with radiotherapy had no effect on improving the survival rate of patients with CSS, and other subgroup analysis showed that surgery could improve the survival time of HCC patients. Multiple studies have also shown that surgery improves survival in patients with different TNM stages and grades.^[32–35]^ In the SEER database, the reasons for patients not undergoing surgery are often described as contraindicated or not recommended, which indicates that these patients are in poor physical condition, insufficient liver reserve function or advanced cancer. For these HCC patients, TACE based interventional therapy is the preferred treatment. Ren study indicates that the combination of TACE with radiofrequency ablation can improve the OS rate in patients with HCC.^[14]^ The use of drug-eluting beads TACE plus camrelizumab has been shown to yield better progression-free survival and tumor response in patients with unresectable HCC.^[36]^ Consequently, TACE is considered the standard for HCC patients who are not candidates for surgery. For patients who can undergo surgery, appropriate surgical treatment should be implemented as early as possible to avoid missing the optimal time for treatment. When necessary, surgery combined with chemotherapy can improve patient survival, which is consistent with previous studies.^[37,38]^

Clinical practice guidelines recommend LR as a first-line treatment for patients with early liver cancer and well-preserved liver function. However, the optimal treatment for patients with HCC with difficult tumor location and advanced cirrhosis who are not suitable for LR remains controversial.^[39,40]^ Wu study only compared the efficacy of surgical and nonsurgical treatment, but did not compare different surgical procedures.^[41]^ To explore the survival rate of patients undergoing different surgical procedures, we divided the procedures into 4 categories and then proceeded to conduct subgroup analysis again. Patients at stages I, II, and III according to the TNM showed the best results for LT, and stage I patients receiving LR had a better prognosis than those receiving WSR. Compared to LTD, LR in stages II and III did not improve patient survival. LT in stage IV patients showed no obvious effect, which may be because advanced patients cannot bear the harm caused by surgical trauma, and the disadvantages of LT outweigh the advantages. Chen showed that the risk of death increases with the age of transplant.^[42]^ Our results should make surgeons aware of the need for risk classification before LT is performed on elderly patients.

The grade subgroup analysis showed that LT was the best treatment in stages I, II, and III and significantly improved survival in both groups, consistent with the findings of Li.^[43,44]^ However, there is a shortage of liver donors, and LT options are limited. Therefore, we should fully realize the importance of organ donation, make better use of the limited number of liver donors, and solve the problem of a mismatch between supply and demand. Our results support priority for LT in patients with early HCC. The OS of patients in stage II showed that LR improved HCC patient outcomes better than WSR, but the CSS outcomes did not show this result, which was similar to the results of several previous studies.^[45–47]^ In stage III, WSR and LR had no significant effect on the prognosis of patients. For patients with poor surgical outcomes or those who experience recurrence after surgery, repeated TACE may be considered for the treatment of localized or intrahepatic residual HCC.^[48]^ Furthermore, the implantation of Iodine-125 seeds following TACE can be used to treat HCC patients with tumors in complex locations, and this therapeutic approach has been shown to effectively improve the OS rate in patients.^[49]^ In summary, for patients with different stages, the preferred surgical method should be carefully considered. Further randomized controlled trials are needed to explore the best treatment model to better guide clinical practice and bring good news to patients.

In addition, we found that chemotherapy combined with WSR and radiotherapy combined with WSR or LR failed to improve the CSS of patients. However, radiotherapy and chemotherapy combined with LT significantly improved the median survival time of patients in both groups. Due to the relatively small number of patients receiving radiotherapy in the database, this can lead to unreliable conclusions. Chemotherapy combined with surgical resection has been found to improve survival in HCC patients,^[50–52]^ consistent with our OS results. However, there are no established clinical guidelines to recommend the use of surgery in combination with chemotherapy in HCC patients. Maybe the potential benefit population is not clear.

In our study, PSM was used to balance the baseline characteristics of the 2 groups of outcomes, reducing the impact of confounding factors on outcomes, and achieving an effect similar to that of randomized controlled studies. Our study also has some limitations. First, this study has a single population source, mostly White people from Europe, and lacks multicenter verification, so its findings cannot be extended to the whole population. Second, the SEER database does not have an exact value for AFP, but only divides AFP into positive and negative groups based on critical boundaries. It may result in the loss of some information. Third, other important information such as vascular infiltration, Child-Pugh score, family history, and postoperative complications are not included in the SEER database, so we are unable to assess the impact of these factors on patient survival in a multivariate analysis.

## Acknowledgments

The authors would like to thank SEER for open access to the database.

## Author contributions

**Conceptualization:** Man Li.

**Data curation:** Lin Xia, Shuai-Xin Yu.

**Formal analysis:** Lin Xia, Yu-Shuai Bai, Fu-Gui Wu.

**Methodology:** Lin Xia, Xiao Liang.

**Writing—original draft:** Lin Xia, Shuai-Xin Yu, Yu-Shuai Bai, Xiao Liang, Fu-Gui Wu, Yang Gao, Xiu-Li Chen, Zhao-Xiong Xiao.

**Writing—review & editing:** Man Li.

## Supplementary Material


