# Supplementary material for: Effect of surgery on overall survival and cancer-specific survival in patients with primary HCC: A study based on PSM in the SEER cohort
Source: Medicine (Baltimore). 2025 Feb 21;104(8):e41521. doi: 10.1097/MD.0000000000041521 (PMC11857015; doi:10.1097/MD.0000000000041521)

Table S1. Variables and classification

Variables	Classification
**Age**	1. ≤60<br>2. 60-70<br>3. >70
**Sex**	1. Female<br>2. Male
**Race**	1. White<br>2. Black<br>3. Other
**Marital**	1. Separated/Divorced<br>2. Married<br>3. Unmarried/Single<br>4. Widowed/Other
**Histotype**	1. HCC others<br>2. HCC NOS
**TNM stage**	1. I<br>2. II<br>3. III<br>4. IV
**T stage**	1. T1<br>2. T2<br>3. T3<br>4. T4
**N stage**	1. N0<br>2. N1
**AFP**	1. Positive<br>2. Negative/Normal
**Surgical method**	1. NO<br>2. YES
**Radiotherapy**	1. NO<br>2. YES
**Chemotherapy**	1. NO<br>2. YES
**Regional nodes**	1. 0<br>2. More than 0
**Primary**	1. NO<br>2. YES
**Grade**	1. I<br>2. II<br>3. III<br>4. IV

*TNM: tumor node metastasis; AFP: alpha-fetoprotein; Primary: first malignant primary indicator

Table S2. Multivariate Cox regression analysis of overall survival and specific survival of HCC before PSM

**Variable name**	**Overall survival of cancer**		**Cancer-specific survival**	
	**HR (95%CI)**	***P* value**	**HR (95%CI)**	***P* value**
**Sex**				
Female	reference		reference	
Male	1.127(1.062-1.196)	<0.001	1.128(1.050-1.213)	0.001
**Grade**				
I	reference		reference	
II	1.163(1.098-1.231)	<0.001	1.182(1.102-1.268)	<0.001
III	1.572(1.468-1.684)	<0.001	1.692(1.559-1.837)	<0.001
IV	1.623(1.351-1.949)	<0.001	1.680(1.354-2.083)	<0.001
**Histotype**				
HCC others	reference		reference	
HCC NOS	0.962(0.807-1.147)	0.666	0.888(0.725-1.089)	0.254
**TNM stage**				
I	reference		reference	
II	1.124(0.923-1.368)	0.246	1.124(0.896-1.411)	0.311
III	1.549(1.335-1.796）	<0.001	1.800(1.515-2.138）	<0.001
IV	2.561(2.244-2.922）	<0.001	2.947(2.527-3.437）	<0.001
**T stage**				
T1	reference		reference	
T2	1.011(0.840-1.216)	0.911	1.083(0.877-1.337)	0.459
T3	1.389(1.212-1.592)	<0.001	1.403(1.199-1.642)	<0.001
T4	1.503(1.272-1.776)	<0.001	1.546(1.278-1.871)	<0.001
**N stage**				
N0	reference		reference	
N1	0.985(0.880-1.103)	0.790	0.940(0.826-1.069)	0.344
**Surgery method**				
No-Surgery	reference		reference	
Surgery	0.268(0.251-0.285)	<0.001	0.247(0.229-0.267)	<0.001
**Radiotherapy**				
No/Unknown/Refused	reference		reference	
YES	0.694(0.643-0.749)	<0.001	0.695(0.636-0.761)	<0.001
**Chemotherapy**				
No/Unknown	reference		reference	
YES	0.694(0.659-0.732)	<0.001	0.730(0.685-0.777)	<0.001
**AFP**				
Negative/Normal	reference		reference	
Positive/elevated	1.336(1.266-1.411）	<0.001	1.508(1.410-1.613)	<0.001
**Region nodes**				
0	reference		reference	
>0	0.718(0.643-0.803)	<0.001	0.712(0.619-0.818)	<0.001
**Primary**				

NO	reference		reference	
YES	0.958(0.899-1.022)	0.191	1.076(0.993-1.165)	0.073
**Race**				
White	reference		reference	
Black	1.039(0.966-1.118)	0.301	1.036(0.949-1.131)	0.431
Others	0.852(0.799-0.908)	<0.001	0.868(0.803-0.937)	<0.001
**Age**				
≤60	reference		reference	
60~70	1.062(1.002-1.127)	0.044	1.064(0.991-1.142)	0.087
>70	1.359(1.274-1.449)	<0.001	1.356(1.255-1.465)	<0.001
**Marital**				
Divorced/Separated	reference		reference	
Married	0.865(0.805-0.930)	<0.001	0.911(0.835-0.994)	0.036
Single/Unmarried	1.018(0.938-1.106)	0.663	1.011(0.905-1.106)	0.989
Widowed	0.946(0.854-1.047)	0.283	0.949(0.838-1.074)	0.406

*HCC: hepatocellular carcinoma; PSM: propensity score matching; TNM: tumor node metastasis; AFP: alpha-fetoprotein; Primary: first malignant primary indicator

Table S3. Multivariate Cox regression analysis of TNM stage, Grade stage, radiotherapy, and chemotherapy in surgery and non-surgery.

Variables		Overall survival of cancer		Cancer-specific survival	
		HR (95%CI)	*P* value	HR (95%CI)	*P* value
**TNM stage**					
	**I**				
	No-surgery	reference		reference	
	Surgery	0.254(0.227-0.285)	<0.001	0.216(0.186-0.250)	<0.001
	**II**				
	No-surgery	reference		reference	
	Surgery	0.318(0.273-0.371)	<0.001	0.311(0.258-0.375)	<0.001
	**III**				
	No-surgery	reference		reference	
	Surgery	0.291(0.248-0.341)	<0.001	0.283(0.235-0.340)	<0.001
	**IV**				
	No-surgery	reference		reference	
	Surgery	0.343(0.253-0.466)	<0.001	0.389(0.276-0.550)	<0.001
**Grade**					
	**I**				
	No-surgery	reference		reference	
	Surgery	0.266(0.230-0.308)	<0.001	0.250(0.207-0.302)	<0.001
	**II**				
	No-surgery	reference		reference	
	Surgery	0.279(0.250-0.311)	<0.001	0.245(0.214-0.281)	<0.001
	**III**				
	No-surgery	reference		reference	
	Surgery	0.317(0.270-0.371)	<0.001	0.330(0.275-0.356)	<0.001
	**IV**				
	No-surgery	reference		reference	
	Surgery	0.168(0.070-0.403)	<0.001	0.148(0.048-0.461)	<0.001
**Radiotherapy**					
	**No/Unknown**				
	No-surgery	reference		reference	
	Surgery	0.277(0.256-0.299)	<0.001	0.260(0.236-0.287)	<0.001
	**Yes**				
	No-surgery	reference		reference	
	Surgery	0.465(0.345-0.628)	<0.001	0.813(0.559-1.182)	0.279
**Chemotherapy**					
	**No/Unknown**				
	No-surgery	reference		reference	
	Surgery	0.236(0.214-0.261)	<0.001	0.220(0.194-0.249)	<0.001
	**Yes**				
	No-surgery	reference		reference	
	Surgery	0.396(0.352-0.445)	<0.001	0.381(0.332-0.438)	<0.001

All

	No-surgery	reference		reference	
	Surgery	0.290(0.269-0.312)	<0.001	0.274(0.250-0.301)	<0.001

*TNM: tumor node metastasis

Fig S1. Love graph before and after PSM

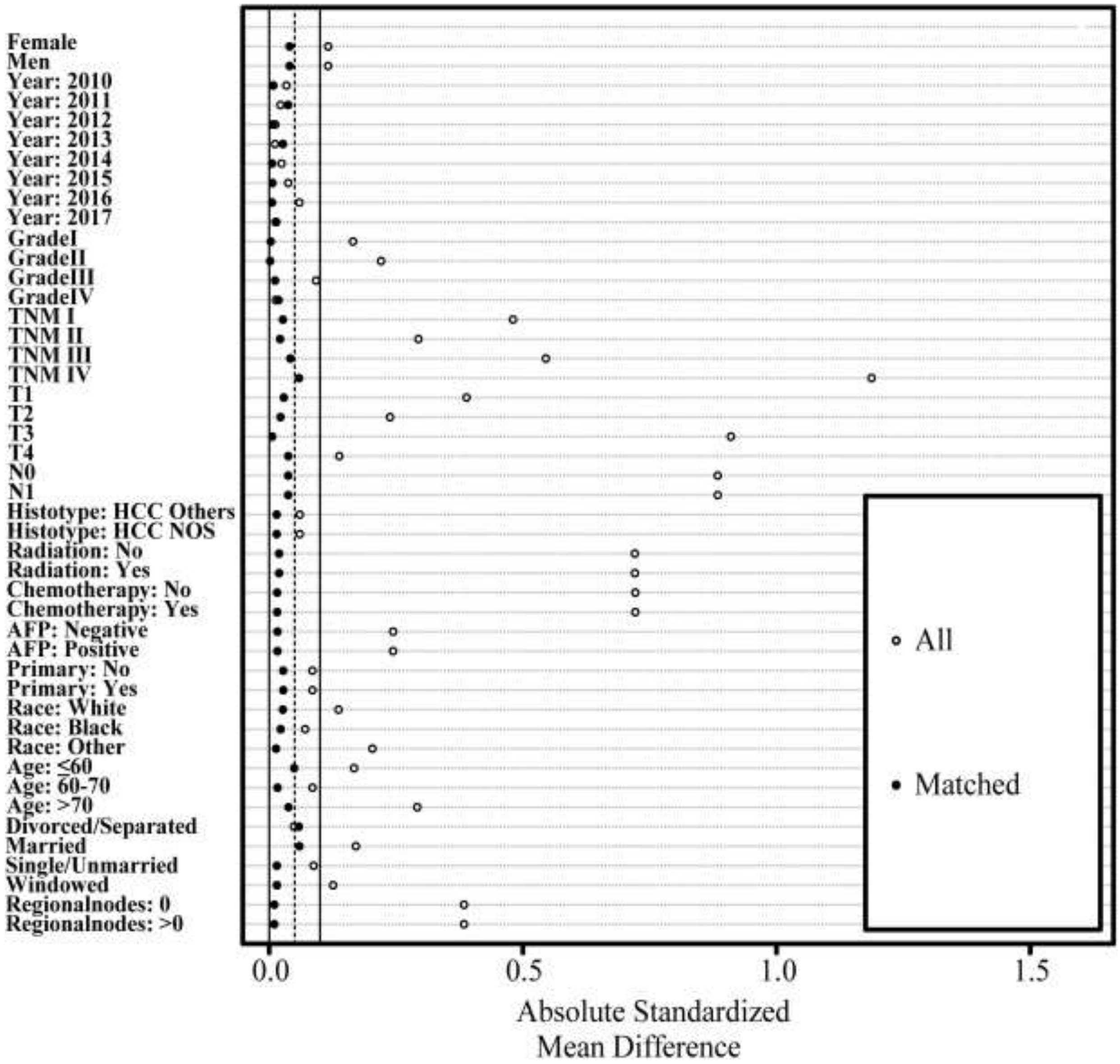

Fig S2. Overall survival (A. Stage I; B. Stage II; C. Stage III; D. Stage IV) and specific survival (E. Stage I; F. Stage II; G. Stage III; H. Stage IV) of patients with HCC in different TNM stages: survival curves of the surgical group and the non-surgical group.

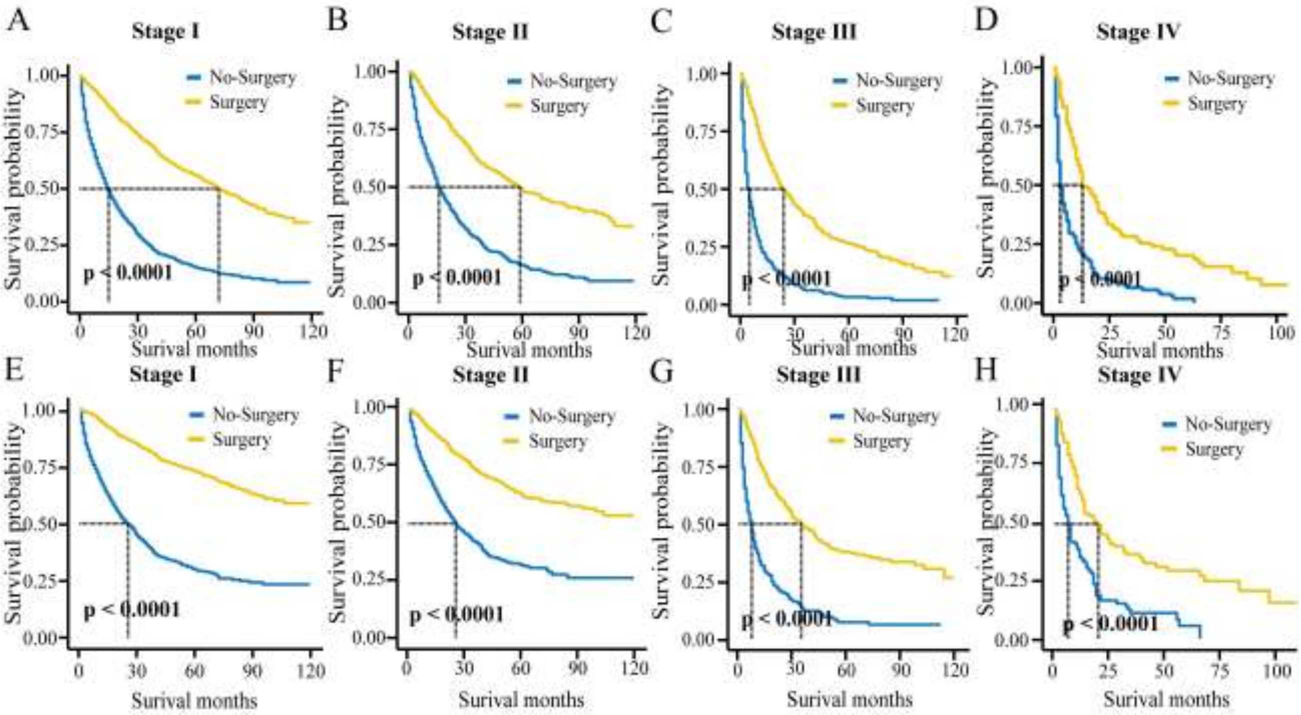

Fig S3. Overall survival (A. Grade I; B. Grade II; C. Grade III; D. Grade IV) and specific survival (E. Grade I; F. Grade II; G. Grade III; H. Grade IV) of patients with HCC in different Grade stages: survival curves of the surgical group and the non-surgical group.

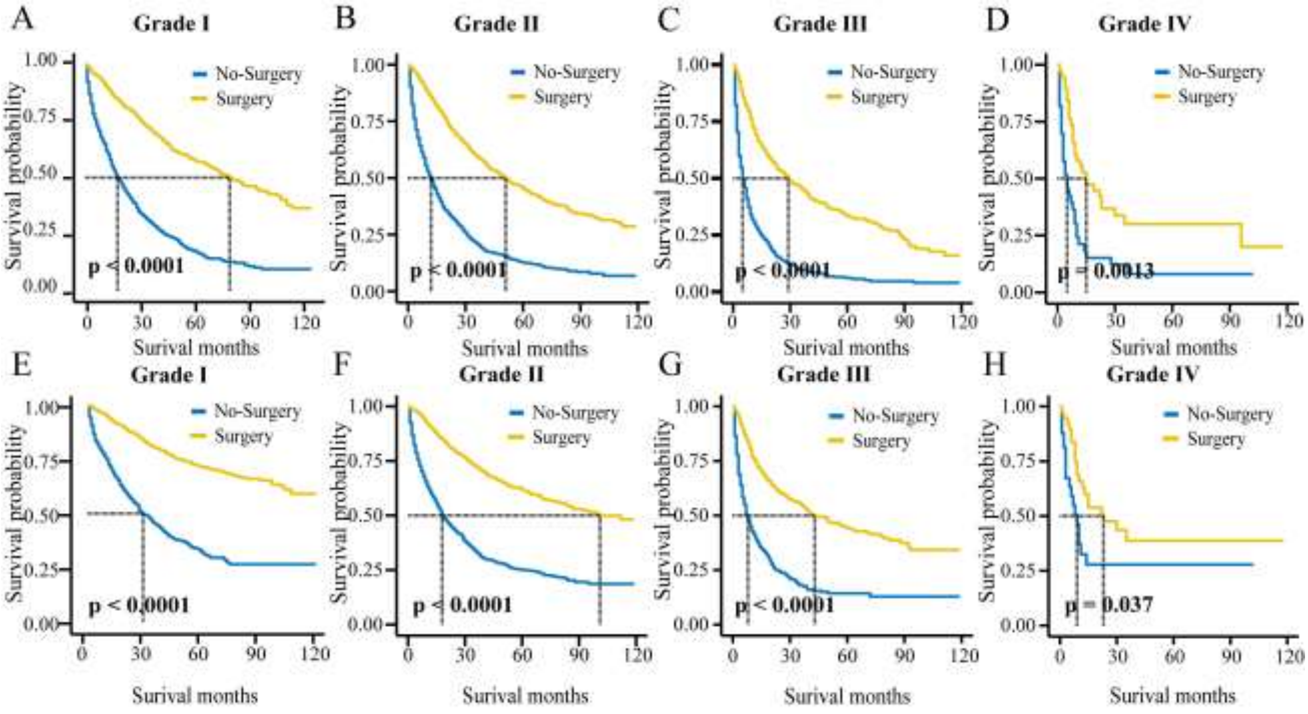

Fig S4. Overall survival (A. No radiotherapy; B. Radiotherapy; C. No chemotherapy; D. Chemotherapy) and specific survival (E. No radiotherapy; F. Radiotherapy; G. No chemotherapy; H. Chemotherapy) curves of patients with radiotherapy and chemotherapy in surgical and non-surgical groups.

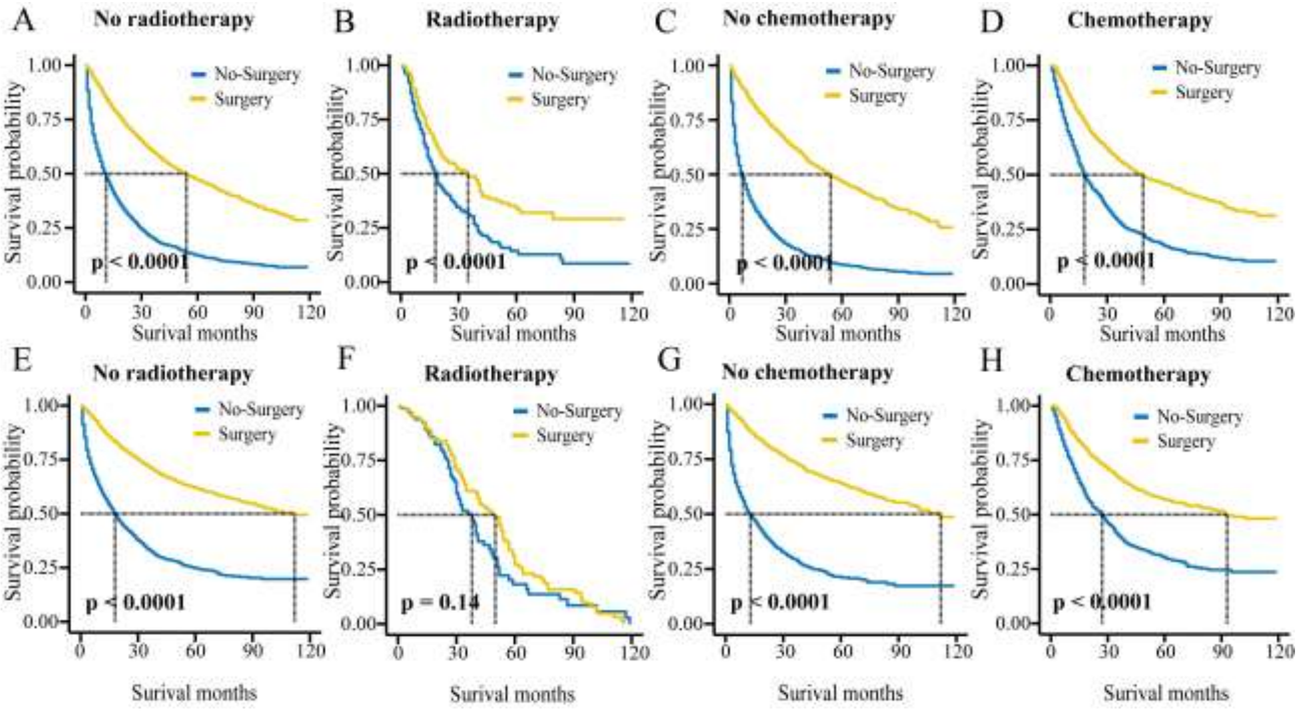

Supplement: Supplementary file 1 [file medi-104-e41521-s001.pdf]
